# Tocilizumab binds to canine IL-6 receptor and elicits *in-vitro* inhibitory biological response

**DOI:** 10.3389/fvets.2025.1645414

**Published:** 2025-09-03

**Authors:** Yael Merbl, Jesus M. Lopez Baltazar, Michael Byron, Sarah K. C. Chan, Jacqueline J. Ehrlich, Qiuming Yu

**Affiliations:** ^1^Department of Clinical Sciences, Cornell University, Ithaca, NY, United States; ^2^Robert Frederick Smith School of Chemical and Biomolecular Engineering, Cornell University, Ithaca, NY, United States; ^3^Department of Molecular Medicine, Cornell University, Ithaca, NY, United States

**Keywords:** tocilizumab, canine, IL-6R, autoimmune, monoclonal antibody

## Abstract

**Introduction:**

Tocilizumab (TCZ) is an immunosuppressive drug approved for the treatment of rheumatoid arthritis in humans. Molecularly, it is a humanized monoclonal antibody (mAb) that binds to the interleukin-6 receptor (IL-6R), blocking its inflammatory pathway with the IL-6 protein. A previous study has looked at the safety of topical TCZ as eye drops in dogs, however, no studies have tested its potential use in canine diseases based on species antibody differences.

**Materials and methods:**

(1) To assess the biological inhibitory effect of TCZ on canine macrophages *in vitro* (*n* = 3), the median fluorescence intensity of phospho-STAT3 (Y705) was determined using flow cytometry and compared to the inhibitory effect of human macrophages (*n* = 2). (2) To try and characterize the receptor region of interest in the canine IL-6R, homology modeling was performed using the MODELLER 10.4 software. (3) To investigate the real-time ligand-binding affinity and kinetic parameters for canine IL-6R with TCZ, surface plasmon resonance (SPR) spectroscopy was used, and results were compared to the human IL-6R interaction with TCZ.

**Results:**

Our results confirm binding of TCZ with canine IL-6R. In comparison, canine IL-6R binds two orders of magnitude less than human IL-6R in its dissociation constant. Canine cell culture required a higher concentration of TCZ compared to human cell culture to produce a similar inhibitory effect.

**Conclusions and clinical significance:**

The binding of TCZ to canine IL-6R resulting in a biological response is a specific example of the new possibilities to harness humanized mAb for canine diseases. TCZ may not be a feasible treatment due to the binding affinity and the high concentrations needed. However, future studies should explore potential suitable human mAb for treating canine autoimmune diseases.

## 1 Introduction

Autoimmunity occurs in both human and veterinary medicine. For decades, traditional therapy has included glucocorticoids as the mainstay, in addition to other immunosuppressive drugs such as mycophenolate mofetil, azathioprine, cyclosporine, and cytosine arabinoside (1, 2). Treatments such as immunoglobulin therapy, plasmapheresis, etc., are becoming the standard of care in some human autoimmune diseases. However, in veterinary medicine similar treatments are far rarer, mainly due to financial constraints and less species-specific research (1, 2). Over the past few decades, monoclonal antibody (mAb) therapy has gained popularity due to its potential as a high specificity blocking molecule, inhibiting downstream events in inflammatory and cancerous diseases. Since the approval of the first mAb by the United States Food and Drug Administration (FDA) in 1986, antibody engineering has markedly advanced (3). Advances in mAb therapy in veterinary medicine are lagging human medicine due to lesser drug development funding availability as well as considerations of market demands. For comparison, the first veterinary mAb for osteoarthritis use was released in 2022 (3, 4), decades after the release of the first mAb for human use. If enough conformational similarity exists in antibody/receptor interaction, harvesting an already approved human mAb for the treatment of domestic animals can shorten the current gap in personalized treatment options.

Tocilizumab (TCZ) is an FDA approved humanized mAb for rheumatoid arthritis and cytokine release syndrome in humans (5), functioning as an interleukin-6 (IL-6) inhibitor by blocking the binding site on the IL-6 receptor (IL-6R).

Prior safety and efficacy trials on dogs using TCZ as a topical eye drop reported no clinical signs to suggest irritation or inflammation in the treated eyes. However, no attempt was made to test whether TCZ should be used in dogs based on Ab specificity (6).

In humans, the IL-6R consists of an Ig-like domain (D1), the cytokine binding module (CBM) domains (D2 and D3), and a 52-amino acid-residue-long flexible stalk region followed by the transmembrane and intracellular domains (7). The IL-6/IL-6R interface is variably conserved between different species, with the canine IL-6R sharing a 74% sequence similarity with humans. A sequence alignment of the D2 and D3 domains of the human and the canine IL-6R revealed a moderate - good conservation between the two species.

When trying to harness a mAb for use in another species, sequence alignments and variable protein conformation between species may impact the potential binding, therefore in the current research we aimed to: (1) investigate the biological response of the drug in canine macrophage cell culture and (2) attempt to characterize the 3-dimensional conformation of the binding site. We hypothesized that TCZ will bind to canine IL-6R and yield an inhibitory biological effect in cell culture.

## 2 Materials and methods

### 2.1 Cell culture and flow cytometry

Under proinflammatory conditions IL-6 binds to the IL-6R, thus triggering a Janus kinase that in turn stimulates the phosphorylation of signal transducer and activator of transcription 3 (STAT3) to initiate downstream signals (8). To look for a biological response we decided to focus on monocytic cell lines, which better resemble cells in the central nervous system (CNS) compared to hepatocytes.

The canine macrophage-like cell line DH82 (CRL-10389) and the human monocyte cell line U-937 (CRL-1593.2) were purchased from the American Type Culture Collection (ATCC, Manassas, VA, USA). DH82 cells were cultured in Eagle’s Minimum Essential Medium (EMEM) supplemented with 15% fetal bovine serum (FBS) and penicillin–streptomycin (ThermoFisher Scientific, Waltham, MA, USA). U-937 cells were cultured in RPMI-1640 supplemented with 10% (FBS) and penicillin–streptomycin. Cells were seeded into 6-well plates and cultured at 37°C and 5% CO_2_. 24 h after seeding, the cells were washed with phosphate buffered saline (PBS) and serum starved for 2 h in serum free media. Cells were incubated with varying concentrations of TCZ (0.1 μg/mL, 10 μg/mL, 500 μg/mL, and 2000 μg/mL) for 30 min, followed by stimulation with 10 ng/mL human IL-6 (for U-937 cells) or 250 ng/mL canine IL-6 (for DH82 cells) for 15 min. Cells were harvested from the plate, centrifuged for 5 min at 125 x g, washed with 2 mL cold PBS with 1% BSA, and then viability was determined using trypan blue exclusion. Cells were then fixed for 30 min at 4°C with BD Cytofix Fixation Buffer (BD Biosciences #554655, Franklin Lakes, NJ), washed twice with PBS with 1% BSA, and permeabilized on ice for 30 min with Perm Buffer III (BD Biosciences). Phosphorylation of the STAT3 protein (p-STAT3) was determined by incubating the cells for 30 min on ice with an RB705 mouse anti-STAT3 (pY705) antibody (BD Biosciences #570663). One sample was stained with an isotype antibody (BD Biosciences #563484) to evaluate non-specific binding. Samples were run on an Attune NxT flow cytometer (ThermoFisher), and results were analyzed using FlowJo software version 10.10. Briefly, forward scatter and side scatter gating was used to exclude debris and doublets from the analysis, and median fluorescence intensity (MFI) was then calculated to determine the level of STAT3 phosphorylation in each group. MFI results were normalized to be expressed as a percentage of the negative control. The experiment was repeated once for the U-937 cells (*n* = 2) and twice for the DH82 cells (*n* = 3).

### 2.2 Sequence alignment and homology modeling

The amino acid sequences of the canine IL-6R (UniProt #A0A8C0PF44) and human IL-6R (UniProt #P08887) and were retrieved from UniProt and aligned using clustal omega.^1^ A structural model of the canine IL-6R D2-D3 domains was generated using MODELLER (9). A crystal structure of the human IL-6R (PDB: IP9M) (10) was used as a reference.

### 2.3 Fluorescence size exclusion chromatography (FSEC) screen

To separate the IL-6R protein for pre-crystallization screening, canine IL-6R alone, TCZ alone, and the IL-6R TCZ complex in a 1:1 molar ratio were run at 0.5 mL/min through a Superdex 200 Increase 10/300 GL column equilibrated with buffer (1X PBS, 5% mannitol, 5% trehalose, 0.01% Tween-80, pH 7.4). Tryptophan fluorescence (280 nm/350 nm) was measured upon elution from the column using HPLC (Shimadzu).

### 2.4 Cryo-EM data collection and analysis

Cryo-EM data collection and analysis is a process that captures high-resolution images of biomolecules in a frozen state, then processes those images to reconstruct a 3D structure of the molecule. Canine IL-6R (XP_855105.1) was combined in 20% excess with TCZ to form a complex by incubating for 10 min on ice. The complex was spotted at 2 mg/mL and 1 mg/mL in 3 ul on glow discharged Quantifoil C-flat Holey Carbon 1.2/1.3 grids and plunge frozen into liquid ethane using a Mark IV FEI Vitrobot. Cryo-EM imaging was performed on a Talos Arctica operated at 200 keV equipped with a K3 direct electron detector and 50 keV gatan imaging filter. Nominal magnification for the collection was 63,000x bringing the super-resolution pixel size to 0.695 Å.

### 2.5 Detection of canine IL-6R and human IL-6R with SPR biosensors

Surface Plasmon Resonance (SPR) experiments were conducted at 25°C using a 6-channel SPR biosensor developed at the Institute of Photonics and Electronics, Prague, Czech Republic. The instrument is equipped with a dispersionless microfluidic system, which allows for the delivery of canine IL-6R and human sIL-6R directly to a TCZ-functionalized chip. A flow cell with six separate flow chambers facing each sensing spot is interfaced with the chip to confine the sample during the experiment. Briefly, the gold chip previously coated with a SAM was mounted into the SPR biosensor. A baseline under MilliQ water at a flow rate of 20 μL/min was established. A TCZ solution with a concentration of 10 μg/mL in SA-10, pH 5.0 was flowed over the surface for at least 15 min at 20 μL/min. Exposure continued until an approximately 70% surface coverage was achieved, corresponding to about 13 nm in wavelength shift. The functionalized surface was briefly washed with SA-10 for 3 min at 20 μL/min, followed by 2 min exposure with PBS 0.5 M NaCl (PBS-Na) at 20 μL/min, removing all noncovalently, loosely bound ligands. A short wash with SA-10 at 20 μL/min followed. The residual unreacted NHS groups were deactivated by injecting an ethanolamine buffer solution 1 M, pH 8.0 for 10 min at 20 μL/min. To quantify the coverage of antibodies immobilized on the surface, the SA-10 buffer was injected again. A typical sensorgram corresponding to the surface functionalization with antibody solutions at various coverages is shown in the Supplementary Figure S1. The thorough instrumentation, setting, reagents, and pre-experiments to compare the binding sites between dogs and humans IL-R and Z are beyond the scope of this manuscript and are all available in the Supplementary file. Three experimental replicates were performed for each measurement.

## 3 Analysis

All analyses were conducted using RStudio version 2025.5.0 (Posit, Boston, MA, USA). For the flow cytometric experiment, a Shapiro–Wilk test was used to test for normality, followed by a one-way ANOVA with a Tukey’s post-hoc test to identify significant differences between groups, with significance set at *p* < 0.05. Binding kinetic data was analyzed using the non-linear global fitting feature.

## 4 Results

### 4.1 Flow cytometry

**Figure 1:**
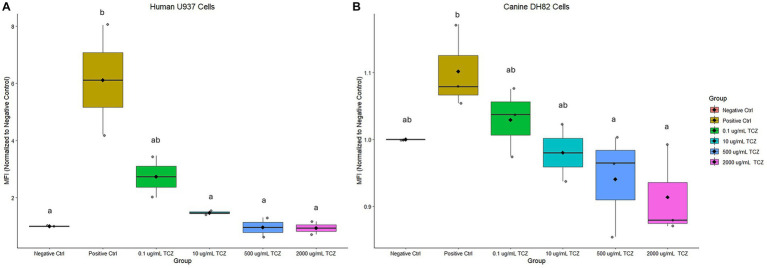
**(A)** Mean fluorescence intensity (MFI) normalized to the negative control. Stimulation of human U937 macrophage cells with 10 ng/mL IL-6 increased STAT3 phosphorylation. Pre-treatment with 10 μg/mL or higher of TCZ significantly decreased STAT3 phosphorylation compared to the positive control. Different letters indicate statistically significant differences between groups (*p* < 0.05) as determined by one-way ANOVA followed by Tukey’s post-hoc test. **(B)** Mean fluorescence intensity (MFI) normalized to the negative control. Pre-treatment of canine DH82 cells with 500 μg/mL or higher of TCZ significantly decreased STAT3 phosphorylation compared to the positive control. Different letters indicate statistically significant differences between groups (*p* < 0.05) as determined by one-way ANOVA followed by Tukey’s post-hoc test.

Human U-937 cells stimulated with human IL-6 (positive control) were found to have an increase in phospho-STAT3 MFI compared to the negative control (Figure 1A, *N* = 2). However, pretreatment with TCZ at a concentration of 10 μg/mL or higher resulted in a decrease in phospho-STAT3 MFI compared to the positive control (*p* < 0.05). Canine DH82 cells (Figure 1B, *N* = 3) were found to require a higher concentration of canine IL-6 (250 ng/mL) to provoke a response in the positive control, and a higher concentration of TCZ was required to significantly diminish phospho-STAT3 MFI in canine cells compared to human cells. Pre-treatment with TCZ at a concentration of 500 μg/mL and 2000 μg/mL resulted in a significant decrease in MFI (*p* < 0.05) in the canine cells compared to the positive control.

### 4.2 Homology modeling

**Figure 2:**
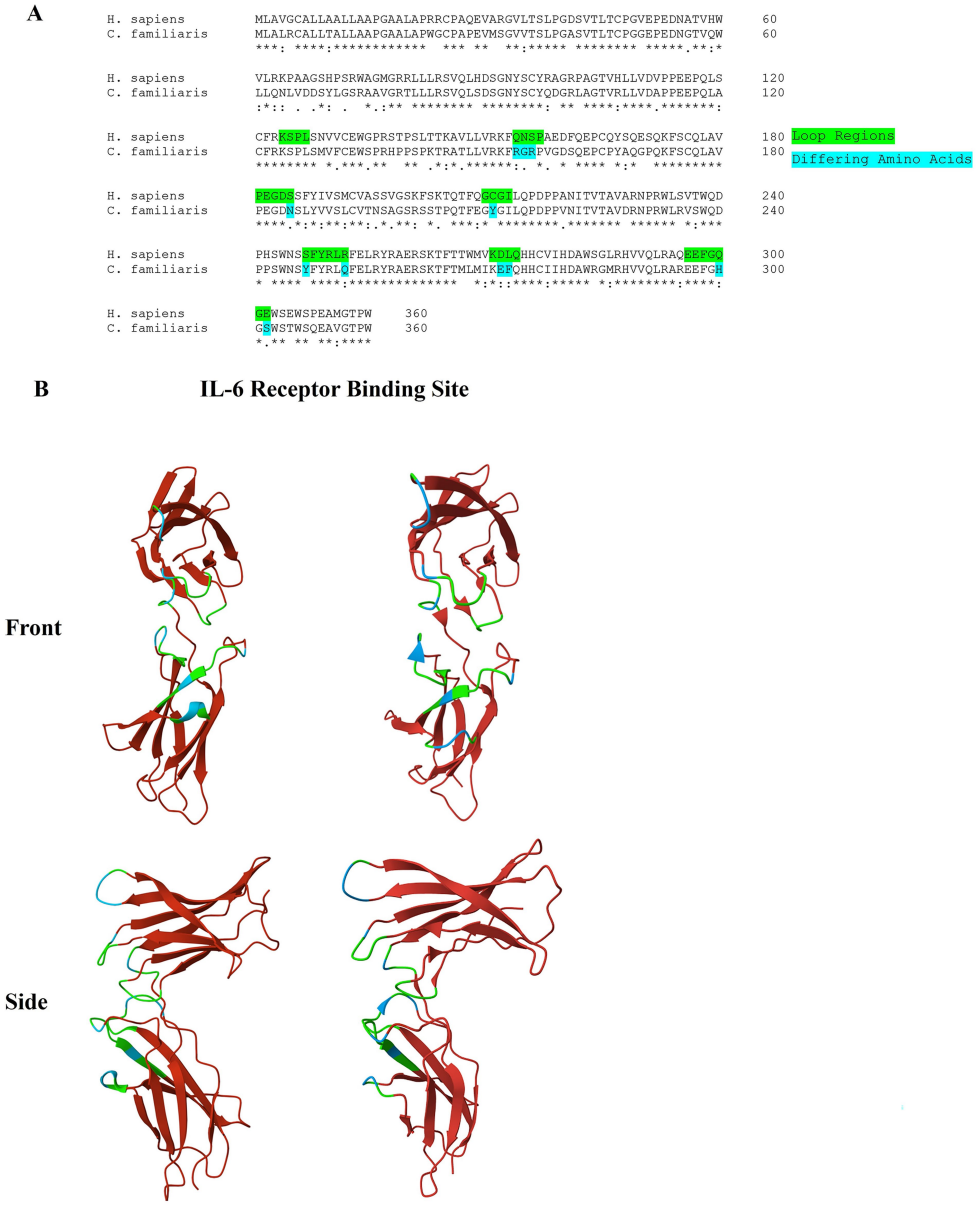
**(A)** Alignment of the amino acid sequence of the human and canine IL-6R. Residues that are found in the loop regions that form the interface for IL-6 binding are highlighted. A ‘*’ symbol indicates a fully conserved amino acid, a ‘:’ symbol indicates a strongly similar amino acid, a ‘.’ symbol indicates a weakly similar amino acid, and a blank space indicates a dissimilar amino acid. **(B)** 3D protein image (front and side view) of the D2 and D3 domains of the human (left) and canine (right) IL-6R. The loop regions that form the interface of IL-6 binding are highlighted in green. Residues that differ from the human IL-6R in these regions are highlighted in blue. A crystal structure of the human IL-6R (PDB: IP9M) was used as a reference.

Homology modeling was performed using the MODELLER 10.4 software with default settings (11). A sequence alignment of the human and canine IL-6R shows some conservation between the two species, with a 74% similarity of the amino acid sequence (Figure 2A). While the specific amino acid residues involved in the interaction between IL-6 and the IL-6R are known in the human, this information has not yet been elucidated in dogs. Figure 2B highlights the loop regions that form the interface involved in binding. Amino acid residues that are different between the human and canine IL-6R in these regions are highlighted blue in the figure. In these loop regions, 67% (23/34) of the amino acids are fully conserved, while 82% (28/34) of the amino acids are either strongly or fully conserved. The differences in these essential areas may potentially impact the capacity of TCZ to bind to the canine receptor.

### 4.3 FSEC supports the complex formation between TCZ and canine IL-6R

**Figure 3:**
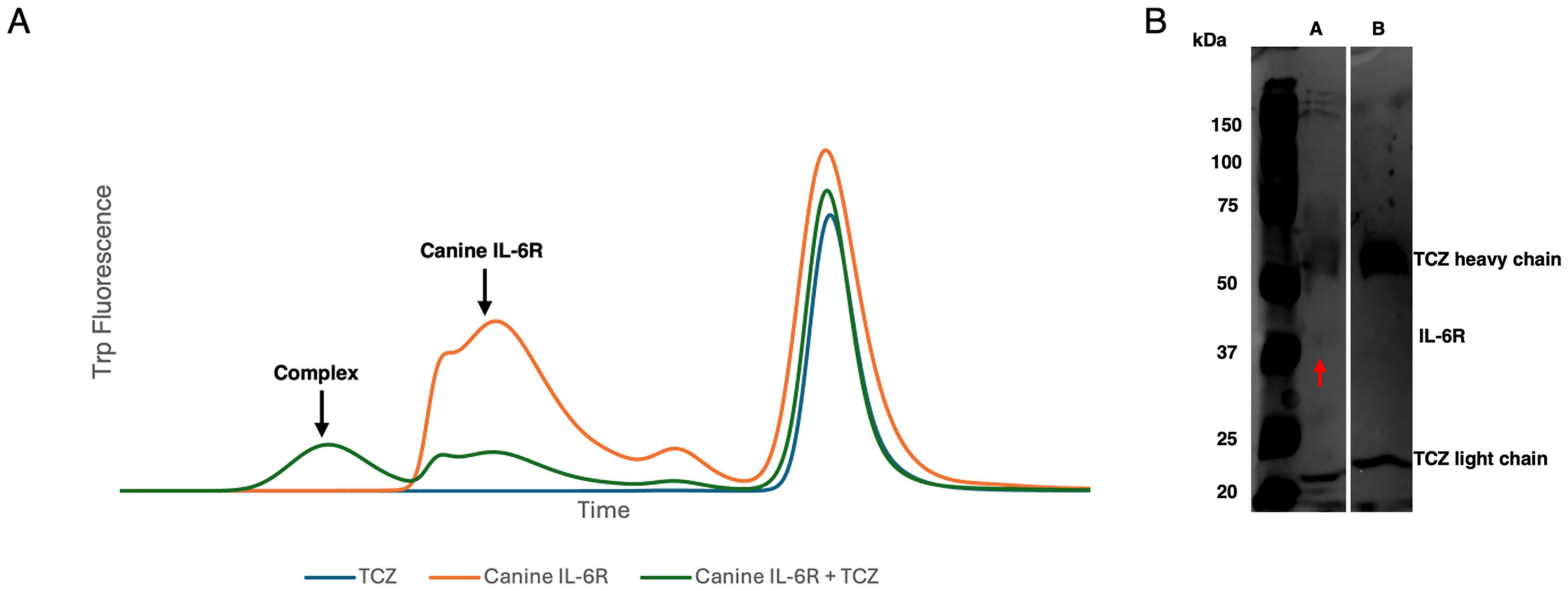
**(A)** FSEC trace of TCZ, canine IL-6R alone, and canine IL-6R TCZ complex. TCZ elutes later than expected likely due to retention on the pre-column filter. **(B)** SDS-PAGE gel of A: 2 mg/mL canine IL-6R + TCZ. B: 1 mg/mL TCZ. The IL-6R is indicated by the red arrow at ~37 kDa in Lane A.

The FSEC approach (12) revealed that the main canine IL-6R peak diminished upon incubation with TCZ and a larger peak appeared shifted to the left, most likely representing the complex formation (Figure 3A). TCZ eluted at a smaller molecular weight later than expected, which could be attributed to loss before reaching the FSEC column: i.e., sticking to the pre-column filter. These results support direct binding of TCZ to canine IL-6R. A reducing SDS-PAGE of the complex showed TCZ breaking into its heavy chain (~50 _k_Da) and light chain (~25 _k_Da). IL-6R appeared at 37 _k_Da (Figure 3B). Given that no other protein was detected in the sample, our FSEC experiments provide support for direct binding of TCZ to canine IL-6R.

### 4.4 Tocilizumab binds to canine IL-6R

**Figure 4:**
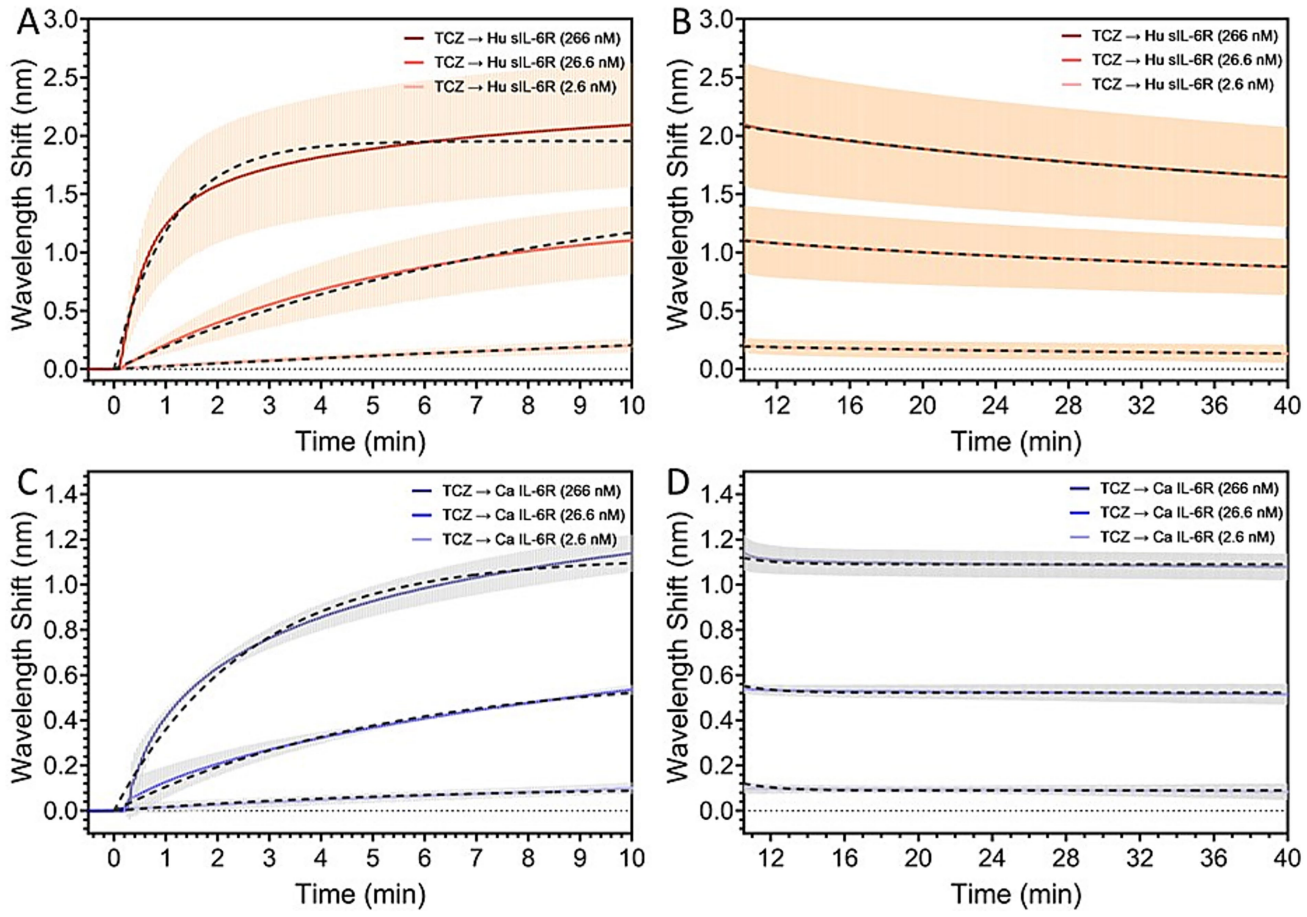
Kinetic fit of the **(A)** association and **(B)** dissociation curves of the binding of human sIL-6R with concentrations of 2.6 nM, 26.6 nM, and 266 nM to the sensor surface functionalized with TCZ. Kinetic fit of the **(C)** association and **(D)** dissociation curves of the binding of canine IL-6R with concentrations of 2.6 nM, 26.6 nM, and 266 nM to the sensor surface functionalized with TCZ. The binding affinity of TCZ to the canine IL-6R is two orders of magnitude weaker than the binding affinity to the human IL-6R.

In our current study, we explored the binding affinity of canine IL-6R compared to the human IL-6R. We found that TCZ binds to canine IL-6R. In a series of analogous experiments, TCZ was immobilized onto SPR sensor chips with surface coverages between 66 and 72% and kept in this range consistently across all replicates. Full results for the SPR analysis can be found in Supplementary file. Kinetic analyses from the SPR Sensorgram indicated a weaker binding affinity of TCZ to the canine IL-6R, with a binding affinity to the human IL-6R that was found to be two orders of magnitude stronger. The dissociation rate K_D_ for the human receptor was found to be 9.9 nM, indicating a strong binding affinity. For the canine receptor, the K_D_ was found to be 203.9 nM, indicating a much weaker binding affinity. Figure 4 depicts the kinetic fit of the (A) association and (B) dissociation curves of the binding of human sIL-6R with concentrations of 2.6 nM, 26.6 nM, and 266 nM to the sensor surface functionalized with TCZ, in comparison to the kinetic fit of the (C) association and (D) dissociation curves of the binding of canine IL-6R with concentrations of 2.6 nM, 26.6 nM, and 266 nM to the sensor surface functionalized with TCZ.

## 5 Discussion

We have explored the biological response *in vitro* of canine and human macrophages to TCZ and have shown that both cell lines produce in a similar biological response, albeit at different concentrations of the mAb. Quantitative kinetic parameters as determined by SPR biosensor data suggested that human sIL-6R interacts with TCZ two orders of magnitude higher compared to canine IL-6R. Homology differences in areas of the canine and human IL-6R that are related to binding may account for the differences seen in binding affinity.

The IL-6 signaling pathway has been shown to play a key role in the dysregulation immune response in a variety of inflammatory diseases (13). Classically, IL-6 binds to the soluble and transmembrane forms of its receptor IL-6R. Gene expression and signaling follow once the complex binds to Glycoprotein 130 (gp130) (14). This pathway has been targeted for the development of drugs, including those which bind to either IL-6 or IL-6R, thus disrupting the inflammatory pathway (7). Numerous studies have shown the high activity of Tocilizumab against the human IL-6R (15, 16). To the best of our knowledge, no studies have reported the binding of Tocilizumab with canine IL-6R. Furthermore, we were able to confirm a biological response in the canine macrophage-like cell line DH82. The human monocyte cell line U-937 was used to confirm the ability of TCZ to block IL-6 signaling in culture. The results of our study are consistent with previously published research (7), which has shown the capacity of TCZ to block IL-6 signaling in U-937 culture through binding of the IL-6R, inhibiting downstream signaling cascades. However, a limited effect was seen in DH82 canine macrophage-like cells when treated with TCZ at similar levels to human U-937 cells. When the concentration of TCZ treatment was increased by 200x, this produced a response in canine cells, with an abatement of phospho-STAT3 MFI similar to the response in human cells at a much lower concentration. These results suggest the binding of TCZ to the canine IL6R, leading to an alteration of the STAT3 pathway and the suppression of downstream signaling. The finding that a much higher concentration of TCZ is needed to provoke this response in culture agrees with the results from the SPR spectroscopy suggesting that TCZ interacts with the canine IL-6R at a level two orders of magnitude lower than with the human IL-6R. While there is no statistical difference indicating that the higher concentrations of TCZ decrease pSTAT3 to levels less than the negative control, it appears that the basal level of STAT3 phosphorylation could have been higher during these experimental conditions. Studies have shown that certain cancer cell lines are more susceptible to serum starvation, leading to an increase in the basal production of IL-6 (17), which could lead to subsequent STAT3 phosporylation. Treatment with TCZ at very high levels might be able to outcompete IL-6 for the IL-6R, leading to a further decrease in STAT3 phosphorylation below the levels of the negative control.

Several limitations should be considered in the current study. Due to time and financial constraints, we were not able to characterize the binding site of TCZ to IL-6R in dogs, and it could differ from the human binding site. We attempted to determine the TCZ binding site for the canine IL-6R using cryo-EM, but representative micrographs showed a spread of aggregated particles, creating a technical barrier (Supplementary Figure S4). A study looking at the conformational state of the IL-6R and TCZ could shed some light on the exact binding site in the dog. The DH82 cell line involved in this study is not composed of naïve cells. Mutation rates in cell lines have been commonly reported as a cause of artifactual results that can produce a response that is different in the live animal. A larger sample size might also be able to better validate the results in future studies.

The exact reason for the species differences in IL-6 potency for phosphorylating STAT3 is unknown. It is possible that there is a difference in the signaling pathways downstream of IL-6 receptor activation. Although transcriptomic analyses indicate that both humans and canines retain the main signaling molecules in the IL-6 pathway, they also reveal significant species-dependent variations in the activated immune transcriptome pathways (18). Thus, it is not surprising to observe differences in IL-6 potency between these species. Another possibility is that the recombinant IL-6 proteins that were purchased had differing specific activities, which is not uncommon for a protein known to decay over time.

In recent years, the treatment for Multiple Sclerosis, an immune mediated CNS disease, has shifted to include monoclonal antibodies modulating immune cell function. Ocrelizumab, a monoclonal antibody against the CD20 antigen on B cells, is an example of such treatment (19, 20). Using SPR spectroscopy biosensors to recognize humanized antibodies that bind to canine proteins may be an easier and more financially feasible route to harness the potential preserved protein receptors.

These results are the first attempt to look at a potential preserved protein receptor, testing TCZ activity on canine IL-6R. Further research is needed looking into the biological safety of the drug and potential therapeutic dosages for TCZ that may not prove realistic, as well as the potential clinical benefits of treatment.

### 5.1 Conclusion

In conclusion, we found evidence that TCZ shows biological activity in canine cells *in vitro*. Compared to the human sIL-6R, TCZ has a two-fold lower binding affinity with the canine IL-6R, with a slower association rate and faster dissociation kinetics by one order of magnitude each. Given the rarity of canine mAb treatments, the harnessing of humanized mAb should be considered for Ab and receptors that conserve their conformation across species and share similar affinity.

## Data Availability

The original contributions presented in the study are included in the article/Supplementary material, further inquiries can be directed to the corresponding author.

## Footnotes

^1^ https://www.ebi.ac.uk/Tools/msa/clustalo/
