# Supplementary material for: Tocilizumab binds to canine IL-6 receptor and elicits in-vitro inhibitory biological response
Source: Front Vet Sci. 2025 Sep 3;12:1645414. doi: 10.3389/fvets.2025.1645414 (PMC12440737; doi:10.3389/fvets.2025.1645414)
Supplement: Supplementary file 1 [file Data_Sheet_1.docx]

**Tocilizumab Binds to Canine IL-6 Receptor and Elicits In-vitro Inhibitory Biological Response. Brief Research Report.**

**Suppelementary information includes:**

1. **Instrumentation**
2. **Reagents**
3. **Preliminary experiments conducted**

**3.1 Self-Assembled Monolayer Formation with Alkane Thiolates**

**3.2 Tocilizumab Immobilization via Amine Coupling Chemistry**

**3.3 SPR Spectroscopy Confirms Binding of Tocilizumab to Human sIL-6R**

**3.4 SPR Spectroscopy Confirms Binding of Tocilizumab to Canine IL-6R**

**3.5. Kinetics Analyses from SPR Sensorgram Show a Weaker Binding Affinity of Canine IL-6R to Tocilizumab compared to Human sIL-6R**

**4. Supplementary figures: Figures S1-S4**

**1. Instrumentation**

Surface Plasmon Resonance (SPR) experiments were conducted at 25 °C using a 6-channel SPR biosensor developed at the Institute of Photonics and Electronics, Prague, Czech Republic. The instrument is equipped with a dispersionless microfluidic system, which allows for the delivery of human sIL-6R and canine IL-6R directly to a TCZ-functionalized chip. A flow cell with six separate flow chambers facing each sensing spot is interfaced with the chip to confine the sample during the experiment. The sensor possesses a temperature controller (ILX Lightwave, LDT-5525) to maintain accurate control in the range of 5 - 40 °C. The incident light to the biosensor chips is collimated and polarized in the sensor head prior to its introduction to the SPR coupling prism. The reflected light is then collected and coupled to the channel spectrographs. For the SPR sensor used in this study, a 1 nm SPR wavelength shift corresponds to a change in protein surface coverage of 17 ng/cm for molecules with a molecular mass of 60 kDa .11 This correlation is then used to calibrate the change in the surface coverage of bound TCZ receptors and IL-6R analytes.

**2. Reagents**

ACTEMRA® (tocilizumab), Genentech. LTD, (South San Francisco, CA) 162 mg injection penfor subcutaneous use was donated by Genentech for the purpose of this study.

Oligo-ethylene-glycol (OEG) alkane thiolates HS-(CH2)11-EG4-OH (Cat. TH 001m11n4-0.2) and HS-(CH2)11-EG6-OCH2-COOH (Cat. TH003m11n6-0.2) were purchased from ProChimia Surfaces (Pomeranian Science and Technology Park, Gdynia, Poland). N-hydroxysuccinimide (NHS) (Cat. 24500) was purchased from Thermofisher Scientific (Waltham, MA, USA). N-(3-Dimethylaminopropyl)-N′-ethylcarbodiimide hydrochloride (EDC) (Cat. 25952-53-8) and sodium chloride (NaCl) ≥ 99.0% were purchased from Millipore Sigma (Burlington, MA, USA). IL-6R Protein, Canine, Recombinant (ECD, His Tag) (Cat. 70117-D08H) was purchased from Sino Biological (Wayne, PA, USA). Recombinant Human sIL-6 Receptor α (CHO derived) (Cat. 200-06RC) was purchased from PeproTech (Cranbury, NJ, USA). Phosphate-buffered saline (PBS), 10X concentrate, pH 7.4 molecular biology grade (Cat. 46-013-CM) was purchased from Corning, Mediatech, Inc (Manassas, VA, USA). Ethanolamine hydrochloride (Cat. 2002-24-6) ≥ 98.0% (by trimetric analysis) was purchased from the Tokyo Chemical Industry Co., Ltd. (TCI) America (Portland, OR, USA). Sodium acetate, anhydrous, 99% (CAS No. 127-09-3) was purchased from BeanTown Chemical, Inc (Sagamore Park Rd, Hudson, NH, USA). Hydrogen Peroxide, 30%, BAKER ANALYZEDTM ACS Reagent Grade (Cat. 2186-01) was purchased from Mallinckrodt Baker, Inc (J.T. Baker) (Phillipsburg, NJ, USA). Sulfuric Acid (Certified ACS Plus) and absolute ethanol 200-proof were purchased from Fisher Scientific (Liverpool, NY, USA).

**3.1 Self-Assembled Monolayer Formation with Alkane Thiolates**

Gold-coated chips were prepared with a 2 nm adhesion promoting chromium layer and a 50 nm active gold thin film via electron-beam evaporation at the Cornell NanoScale Facility (CNF). The chips were then washed with a 3:1 H2SO4:H2O2 piranha mixture for 5 min to remove any organic impurities on the surface. Afterwards, the chips were rinsed with 18.2 MΩcm MilliQ water, and ethanol, drying with nitrogen. The bare gold surface was further subject to UV ozone treatment for 5 min and subsequently rinsed with MilliQ water, and ethanol, drying with nitrogen. Then the chip surface was functionalized with a mixed self-assembled monolayer (SAM) by incubation with -OH terminated (EG4OH) and -COOH terminated (EG6COOH) thiols at a 7:3 molar ratio. The chip immersion was performed overnight and at room temperature in a 0.2 mM ethanol solution mixture of EG4OH and EG6COOH. The 7:3 ratio was used to maximize TCZ binding while minimizing non-specific interactions.

**3.2 Tocilizumab Immobilization via Amine Coupling Chemistry**

TCZ was immobilized on a mixed SAM functionalized gold chip surface using amine coupling chemistry. Briefly, the gold chip previously coated with a SAM was mounted into the SPR biosensor. A baseline under MilliQ water at a flow rate of 20 µL/min was established. The carboxylate groups from the EG6COOH thiols were activated by exposure of a pre-prepared mixture of NHS (20 mM) and EDC (80 mM) in MilliQ water for 10 min at a flow rate of 5 µL/min, followed by MilliQ wash at 20 µL/min. Sodium acetate buffer (10 mM) (SA-10), pH 5.0 was introduced to obtain a stable baseline. A TCZ solution with a concentration of 10 µg/mL in SA-10, pH 5.0 was flowed over the surface for at least 15 min at 20 µL/min. Exposure continued until an approximately 70% surface coverage was achieved, corresponding to about 13 nm in wavelength shift. The functionalized surface was briefly washed with SA-10 for 3 min at 20 µL/min, followed by 2 min exposure with PBS 0.5 M NaCl (PBS-Na) at 20 µL/min, removing all noncovalently, loosely bound ligands. A short wash with SA-10 at 20 µL/min followed. The residual unreacted NHS groups were deactivated by injecting an ethanolamine buffer solution 1M, pH 8.0 for 10 min at 20 µL/min. To quantify the coverage of antibodies immobilized on the surface, the SA-10 buffer was injected again. A typical sensorgram corresponding to the surface functionalization with antibody solutions at various coverages is shown in Figure S1.

To measure the binding kinetic parameters of Hu sIL-6R and Ca IL-6R to TCZ, the running buffer was flown for at least 30 min post sample detection. The final detection levels were assessed as the difference in the wavelength shift from the sensor response between the buffer baselines before sample injection, and after washing the reacted surface with PBS (1X) buffer. The running buffer for the detection step was PBS (1X), pH 7.4. Human (Hu) sIL-6R and canine (Ca) IL-6R sample solutions with concentrations of 2.6 nM, 26.6 nM, and 266 nM, each, were flown in PBS (1X) over the TCZ-functionalized chip at 20 µL/min. Running PBS (1X) buffer followed at the standard flow rate of 20 µL/min, which was the same used to establish the baseline.

**3.3 SPR Spectroscopy Confirms Binding of Tocilizumab to Human sIL-6R**

TCZ was immobilized onto SPR chips with surface coverages between 66 – 71%. Surface coverage was kept consistent across all experiments, promoting optimal interaction with the sIL-6R. As expected, TCZ binds to human sIL-6R. Figure S2(A) and (B) show the SPR sensorgrams and wavelength shifts of three experimental replicates for the interaction of human sIL-6R (266 nM) with TCZ. The detection levels, in this case, fell between 1.2 – 2.1 nm in wavelength shift. The experimental replicates performed for the human sIL-6R at concentrations of 26.6 nM and 2.6 nM are shown in Figure S2. The wavelength shifts and SPR sensorgram are shown for each replicate. The detection levels of human sIL-6R with TCZ were between 0.6 – 1.1 nm in wavelength shift for the 26.6 nM concentration case. The levels of detection for the concentration of 2.6 nM of human sIL-6R interacting with TCZ corresponded to 0.05 – 0.20 nm in wavelength shift. For each concentration of human sIL-6R, the average of all experimental replicates is shown in the SPR sensorgram in Figure S2(C). Error bars were also included. The final detection curve for human sIL-6R interacting with TCZ at concentrations of 266 nM, 26.6 nM, and 2.6 nM is shown in Figure S2(D).

**3.4 SPR Spectroscopy Confirms Binding of Tocilizumab to Canine IL-6R**

The binding affinity of canine IL-6R was also investigated. Interestingly, TCZ binds to canine IL-6R. In a series of analogous experiments, TCZ was immobilized onto SPR sensor chips with surface coverages between 66 – 72% and kept in this range for consistency across all replicates. Figure S3(A) and (B) shows the SPR sensorgrams and wavelength shifts of three experimental replicates for the interaction of canine IL-6R (266 nM) with TCZ. The detection levels fell between 1.05 – 1.1 nm in wavelength shift. The experimental replicates performed for the canine IL-6R at concentrations of 26.6 nM and 2.6 nM were also performed and are shown in Figure S3. The detection levels of canine IL-6R (26.6 nM) with TCZ were between 0.45 – 0.55 nm in wavelength shift. Furthermore, the detection levels for the canine IL-6R (2.6 nM) with TCZ were between 0.05 – 0.12 nm in wavelength shift. Figures 3(C) and (D) show the average SPR sensorgrams and detection curve for all concentrations. For the canine species, the detection levels fell in a narrower and lower range compared to the human case. However, binding with TCZ is evident.

**3.5. Kinetics Analyses from SPR Sensorgram Show a Weaker Binding Affinity of Canine IL-6R to Tocilizumab compared to Human sIL-6R**

A direct target-ligand binding assay model describes the interaction of TCZ to human sIL-6R and canine IL-6R to form a TCZ – IL-6R complex (Eq. 1).

$$\begin{aligned} \left[ TCZ \right]+\left[ IL\_6R \right]\begin{matrix} k_{a} \\ \leftrightarrow\\ k_{d} \end{matrix}[TCZ-IL\_6R]\#\left( 1 \right) \end{aligned}$$

The kinetic parameters such as the association rate k_a_, the dissociation rate k_d_, and the equilibrium dissociation constant *K_D_=k_d_ ⁄ k_a_* were estimated from the SPR sensorgrams obtained during the detection step. A non-linear global fitting was performed to the resulting signals obtained for each of the concentrations used (2.6 nM, 26.6 nM, and 266 nM). The value of *k_d_* was estimated from the dissociation phase while *k_a_* was estimated from the association phase. Figures 4(A) and (B) of the main manuscript show the association and dissociation phases for human sIL-6R interacting with TCZ. Likewise, Figures 4(C) and (D) shows the corresponding association and dissociation phases for canine IL-6R interacting with TCZ. The fitting curves are over imposed on each of the plots. Table S1 shows the calculated binding kinetic parameters for each case.

A lower *K_D_* indicates a greater binding affinity. For the human receptor, the estimated *k_a_* and *k_d_* were 5.5 × 104 M^-1^·s^-1^ and 5.5 × 10-4 s^-1^ respectively. The corresponding *K_D_* value was 9.9 nM indicating a strong binding affinity. Analogously, for the canine receptor, the estimated *k_a_* and *k_d_* were 1.3 × 104 M^-1^·s^-1^ and 2.6 × 10-3 s^-1^ respectively. The corresponding *K_D_* value was 203.9 nM which in comparison is two orders of magnitude weaker than the human receptor’s binding affinity because of the slower association rate and faster dissociation rate of canine IL-6R binding to TCZ.

**Table S1.** Quantitative kinetic parameter of TCZ binding to human and canine IL-6R

| Interaction | $\boldsymbol{k}_{\boldsymbol{a}}$ [M-1·s-1] | $\boldsymbol{k}_{\boldsymbol{d}}$ [s-1] | $\boldsymbol{K}_{\boldsymbol{D}}$ [nM] |
| --- | --- | --- | --- |
| TCZ – Hu sIL-6R | 5.5 × 104 | 5.5 × 10-2 | 9.9 |
| TCZ – Ca IL-6R | 1.3 × 104 | 2.6 × 10-1 | 203.9 |

**Supplementary Figures:**


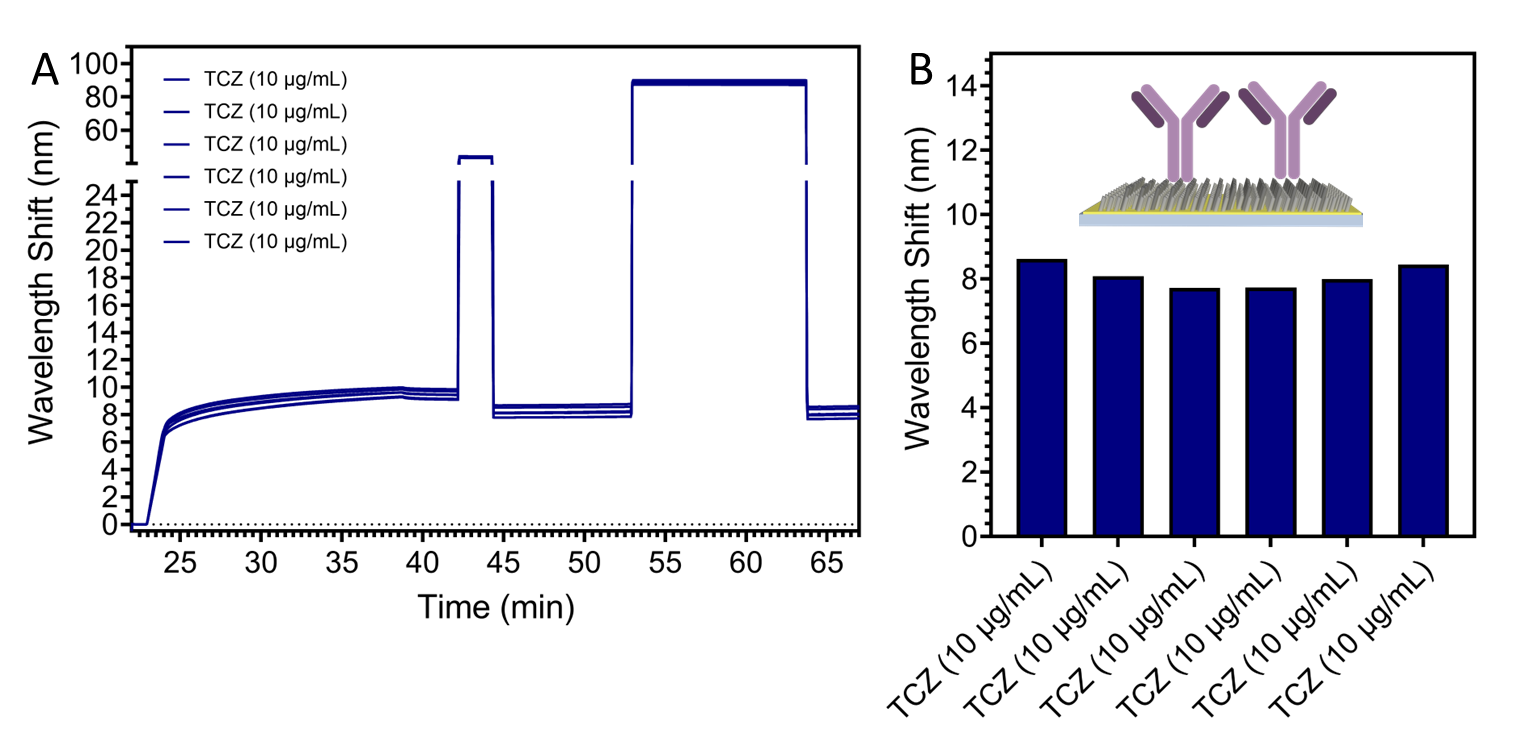


**Figure S1.** (A) SPR sensorgram and (B) wavelength shift detection levels showing the binding of TCZ onto a SPR chip substrate coated with a EG4OH:EG6COOH SAM at a 7:3 ratio. The surface was previously activated using NHS/EDC.


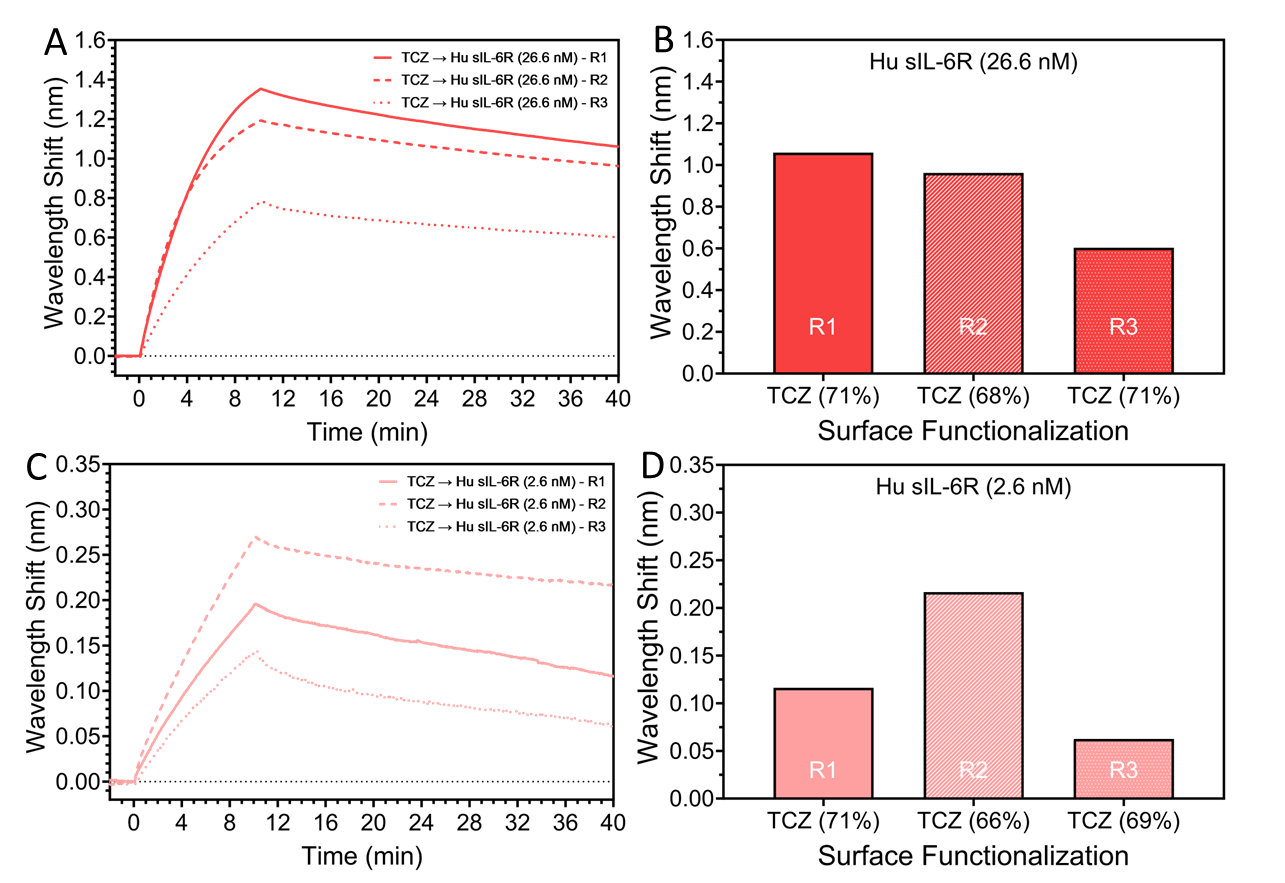


**Figure S2.** (A) SPR sensorgram and (B) wavelength shift detection levels showing three different experimental replicates (denoted as R1, R2, and R3) of the interaction of human sIL-6R (26.6 nM) with TCZ-functionalized chips. The TCZ coverage ranged from 68% to 71% across replicates. (C) SPR sensorgram and (D) wavelength shift detection levels showing three different experimental replicates of the interaction of human sIL-6R (2.6 nM) with TCZ-functionalized chips. The TCZ coverage ranged between 66% to 71% across replicates.


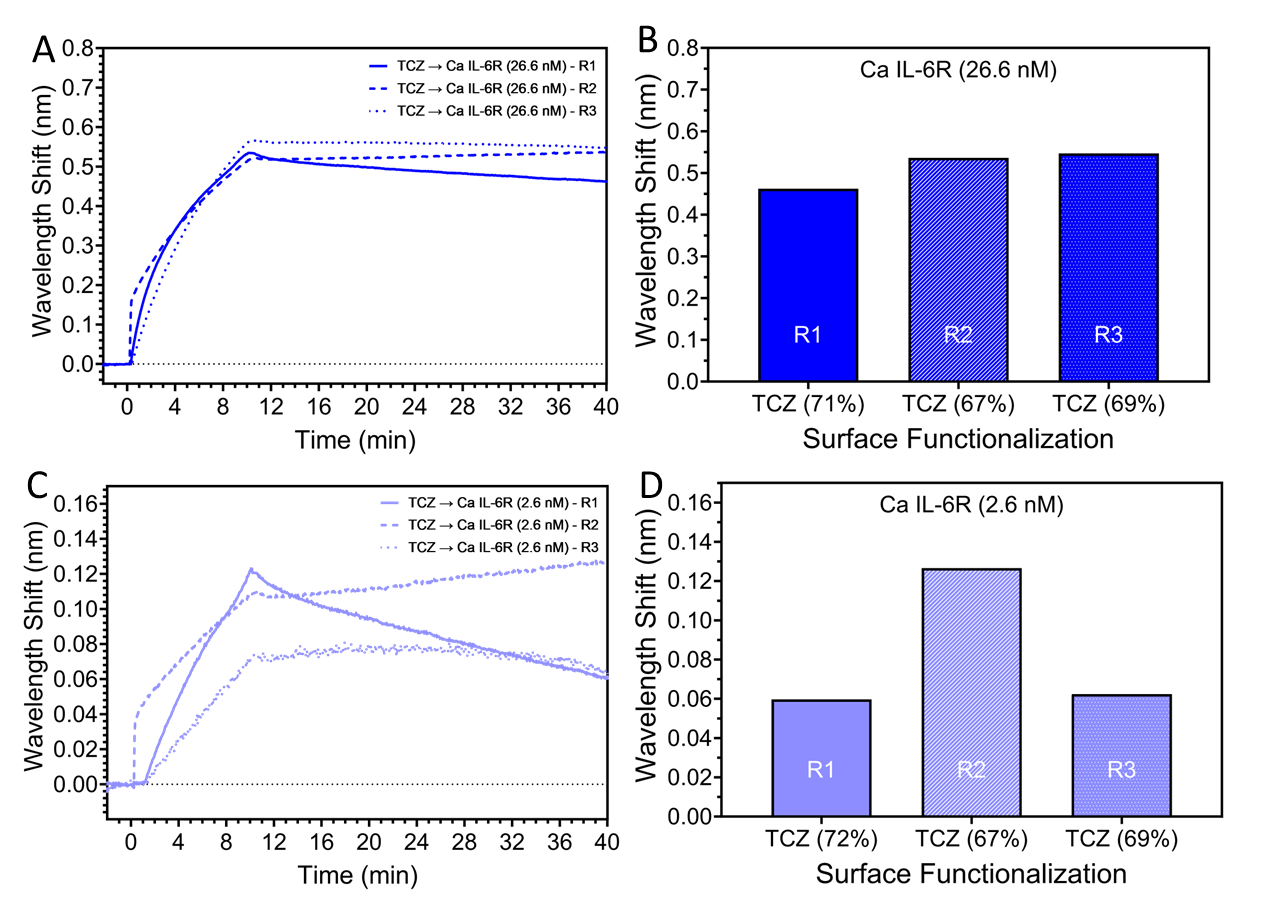


**Figure S3.** (A) SPR sensorgram and (B) wavelength shift detection levels showing three different experimental replicates (denoted as R1, R2, and R3) of the interaction of canine IL-6R (26.6 nM) with TCZ-functionalized chips. The TCZ coverage ranged from 67% to 71% across replicates. (C) SPR sensorgram and (D) wavelength shift detection levels showing three different experimental replicates of the interaction of human sIL-6R (2.6 nM) with TCZ-functionalized chips. The TCZ coverage ranged between 67% to 72% across replicates.

**
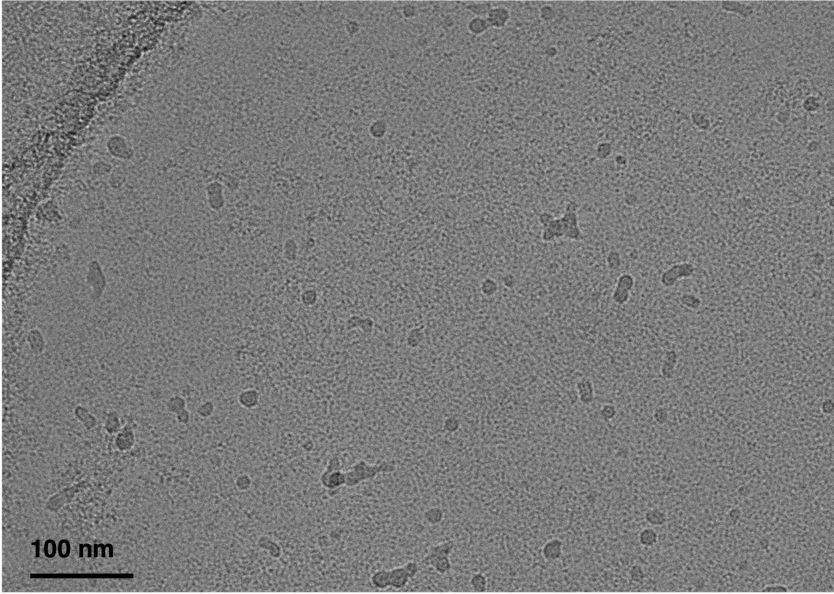
**

**Figure S4.** Micrograph of canine IL-6R TCZ complex at 2 mg/ml without discernable protein particles.
